# Sxt1, Isolated from a Therapeutic Phage Cocktail, Is a Broader Host Range Relative of the Phage T3

**DOI:** 10.3390/v16121905

**Published:** 2024-12-11

**Authors:** Polina Iarema, Oksana Kotovskaya, Mikhail Skutel, Alena Drobiazko, Andrei Moiseenko, Olga Sokolova, Alina Samitova, Dmitriy Korostin, Konstantin Severinov, Artem Isaev

**Affiliations:** 1 Center for Molecular and Cellular Biology, Moscow 121205, Russia; polinaiarema@gmail.com (P.I.); ktv.xna@gmail.com (O.K.); mskutel@yandex.ru (M.S.); alena.drobiazko.brex@gmail.com (A.D.); 2 Faculty of Biology, Lomonosov Moscow State University, Moscow 119991, Russia; postmoiseenko@gmail.com (A.M.); sokolova@mail.bio.msu.ru (O.S.); 3 Center for Precision Genome Editing and Genetic Technologies for Biomedicine, Pirogov Russian National Research Medical University, Moscow 117997, Russia; alinasamitova16@gmail.com (A.S.); d.korostin@gmail.com (D.K.); 4 Institute of Gene Biology Russian Academy of Sciences, Moscow 119334, Russia; severik@waksman.rutgers.edu

**Keywords:** bacteriophage T3, bacteriophage T7, Sextaphage, phage therapy, lateral tail fibers, ejectosome

## Abstract

Using *Escherichia coli* BW25113 as a host, we isolated a novel lytic phage from the commercial poly-specific therapeutic phage cocktail Sextaphage^®^ (Microgen, Russia). We provide genetic and phenotypic characterization of the phage and describe its host range on the ECOR collection of reference *E. coli* strains. The phage, hereafter named Sxt1, is a close relative of classical coliphage T3 and belongs to the *Teetrevirus* genus, yet its internal virion proteins, forming an ejectosome, differ from those of T3. In addition, the Sxt1 lateral tail fiber (LTF) protein clusters with those of the phages from the *Berlinvirus* genus. A comparison of T7, T3, and Sxt1 LTFs reveals the presence of insertions leading to the elongation of Sxt1 tail fibers, which, together with the difference in the HRDRs (host range-determining regions), might explain the expanded host specificity for the Sxt1.

## 1. Introduction

Faced with an overall rise in microbial resistance to antibiotics, researchers across the globe are looking for alternative therapies to treat bacterial infections [1]. Bacteriophages, or phages, are natural predators of bacteria. Given their high selectivity for microbial hosts and relatively rare adverse effects in the human body [2], phages are considered to be one of potential solutions to the antibiotic resistance crisis [3,4,5,6,7]. Yet, phage therapy finds limited use, mostly as a personalized medicine against pathogens isolated from patients who do not respond to antibiotic treatment [8,9,10]. Clinical trials have been performed to access the therapeutic potential of phages for treatment of *Pseudomonas aeruginosa*-infected surface wounds (topical administration) [11], infant diarrhea caused by enteropathogenic *E. coli* (oral administration) [12], and *Staphylococcus aureus* bacteremia (by intravenous injection) [13]. These reports demonstrated the safety of phage treatments and the principal possibility to target bacterial pathogens in situ. At the same time, phage therapy faces many challenges, such as achieving active phage replication in the patient’s body after administration, standardization of phage preparations, and scaling of phage production. Another problem is the rapid development of resistant bacterial strains through mechanisms such as surface receptors change or production of capsule or O-antigens, which prevent phage adsorption [14,15,16]. To overcome this quandary, phages with extended host range and “relaxed” receptor specificity are required.

Polyvalent phage cocktails, containing a mixture of uncharacterized phages selected from natural sources, are commercially available in some countries. For example, Eliava BioPreparations (Georgia) and Microgen (Russia) produce phage cocktails for internal or external administration [17,18,19,20]. Often, the phage composition of these mixtures is not revealed, and therapeutic phages remain uncharacterized on the genomic level, although some studies applied metagenomic approaches to describe the diversity of phages in commercial preparations [21,22,23,24]. The polyvalent Sextaphage^®^ (Microgen, Russia) contains phages isolated on *Staphylococcus spp*., *Streptococcus* spp., *Proteus* (*Р. vulgaris*, *P. mirabilis*), *P. aeruginosa*, *Klebsiella pneumoniae*, and enteropathogenic *E. coli* and was used in a few medical studies [25,26,27,28]. Using Sextaphage^®^ as a source we isolated a new phage infecting model laboratory *E. coli* strain BW25113. The new phage, a podovirus that we named Sxt1, is a close relative of the phage T3. However, Sxt1 is able to propagate on *E. coli* strains that are not infected by T3. Analysis of the Sxt1 genome and AlphaFold modeling revealed distinct organization of the Sxt1 lateral tail fiber (LTF) protein, which may be responsible for its expanded host range. In addition, Sxt1 differs from T3 in the protein module composing ejectosome, the molecular machine that cleaves the peptidoglycan layer, forms a channel across the periplasm, and injects the phage genome into the cytoplasm [29,30], which may also contribute to its expanded host range.

## 2. Materials and Methods

### 2.1. Bacteria, Phages, and Growth Conditions

The following *E. coli* strains were used in this work: BW25113, MG1655, BL-21, B, C, DH5a, HS, Nissle1917, BW39773, KD263, and ECOR (72 strains). Bacteria were routinely propagated in liquid LB media (m/v: 10% Triptone, 5% Yeast extract, 10% NaCl) at 37 °C. Bacteriophages T7 (*Teseptimavirus T7*) and T3 (*Teetrevirus T3*) were from the laboratory collection and were propagated as described below.

### 2.2. Sxt1 Bacteriophage Isolation and Purification

Sxt1 was isolated from the polyvalent phage cocktail Sextaphage^®^ (Microgen, Perm, Russia, batch P239, issued 04/2022) via addition of 10 μL of the phage mix to the top 0.6% LB agar with *E. coli* BW25113 culture. Single plaques were re-streaked and then inoculated into 10 mL of bacterial culture pre-grown in LB to OD_600_ = 0.3 (~10^8^ cfu/mL). Phage infection was carried out overnight at 37 C. Cell lysate was spun down by centrifugation (10 min at 6000× *g*) and treated with 50 µL of chloroform before storage.

For downstream genomic DNA extraction, the phage was precipitated with PEG. In short, 8 mL of lysate with titer ~10^10^ pfu/mL was treated with 2 µL DNAse I at 37 °C for 30 min to remove fragments of host DNA, and then was mixed with 2 g of PEG 8000; NaCl was adjusted in solution to 1 M. Phage particles were precipitated at +4 °C overnight with rotation and then collected by centrifugation (10 min at ~3600× *g* in bucket rotor). Precipitate was resuspended in 500 µL of STM buffer (NaCl—100 mM, MgSO_4_—10 mM, Tris-HCl, and pH = 7.5–50 mM). To remove PEG, 500 µL of chloroform was added and mixture was rigorously vortexted for 1 min. Following centrifugation (5 min at 6000× *g*), the supernatant was collected and stored at 4 °C.

For transmission electron microscopy and cryo-electron microscopy, Sxt1 lysate was concentrated and purified using a sucrose cushion, as described [31]. In total, 30 mL of high-titer phage lysate (~10^10^ pfu/mL) was layered on top of a 38% (*w*/*v*) sucrose cushion prepared using 0.2 mm filtered SM buffer (50 mM Tris-HCl pH 7.5, 100 mM NaCl, and 8 mM MgSO_4_) at a ratio of one part sucrose to seven parts viral concentrate. The tubes were centrifuged for three hours at 15 °C in a P40ST rotor (CP100NX, Himac, Hitachinaka, Japan) at 32,000 rpm (181,000× *g*). The pellet under the sucrose cushion was gathered in 100 µL of Tris-EDTA buffer (TE, 10 mM Tris-HCl pH 7.6, 1 mM EDTA).

### 2.3. Transmission Electron Microscopy

For negative staining transmission electron microscopy (TEM), the formvar/carbon Cu-supported TEM grid (Ted Pella, catalog number 01801) was cleaned in Ar: O_2_ plasma for 40 s (1070 Nanoclean, Fischione, PA, USA). A volume of 20 µL of phage lysate was dropcasted onto the carbon side of the grid and left for 1 min. The residual sample was blotted by touching the grid with the blot paper, followed by two rinses in droplets of distilled H_2_O. After that, the grid was immediately floated on top of the drop of uranyl acetate (UA, 1 wt.% solution, 9 µL) and was held in touch with UA droplet with tweezers for 45 s. The excess negative stain was blotted by gently sliding the side of the grid along the piece of blotting paper. The grid with the stained sample was left in the air until completely dry.

Bright-field TEM images were acquired on a Titan Themis Z transmission electron microscope (Thermo Fisher Scientific, Bleiswijk, The Netherlands) operated at 200 kV using a BM-Ceta 4 K × 4 K CMOS camera with 4-pixel binning.

### 2.4. Cryo-Electron Microscopy

For the cryo-electron microscopy studies, a suspension of 3 µL of the sucrose cushion purified sample was applied to a lacey carbon support film mounted on a 300 mesh copper grid (Ted Pella). Prior to application, the grid was glow-discharged in residual air at a pressure of 0.26 mbar with a current of 25 mA for 30 s using an EasyGlow device (Pelco, Fresno, CA, USA). The sample was maintained on the grid surface for 30 s, followed by blotting from the carbon side for 8 s with Whatman #1 filter paper, and was then rapidly frozen in liquid ethane at −180 °C (EM GP2, Leica Microsystems, Wetzlar, Germany).

CryoEM data were collected using a JEM-2100 (JEOL, Showima City, Tokyo, Japan) transmission electron microscope operating at 200 kV and equipped with a DE20 (Direct Electron, San Diego, CA, USA) camera. A total of 1000 images were acquired with a defocus of 3 µm, a pixel size of 2.31 Å, and a total dose of 50 e/Å^2^. The images underwent processing using CryoSPARC v.4.0 software. After performing patch motion correction and CTF estimation, 15,000 virion projections (particles) were picked and selected for 3D reconstruction. The initial 3D reference was generated using an ab initio algorithm and subsequently refined through homogeneous refinement with icosahedral symmetry. The icosahedral capsid reconstruction yielded the resolution of 14 Å.

The particles were then subjected to symmetry expansion based on icosahedral symmetry, followed by 3D classification using a mask that included one of the capsid vertices. Particles from classes exhibiting tail density in the class averages were further refined in 3D with C6 symmetry to reconstruct the tail density. The final reconstruction achieved a resolution of 16 Å, measured using the FSC 0.143 criterion.

### 2.5. One-Step Growth Curve

One-step growth curves assays were performed with *E. coli* BW25113 pre-grown in 10 mL LB to OD_600_ = 0.6 (~2 × 10^8^ cfu/mL). Sxt1 or T7 from a 10^10^ pfu/mL stock were added to achieve an initial Multiplicity of Infection (MOI) of 0.001. An amount of 1 mL aliquot was collected every 10 min and centrifuged for 5 min at 6000× *g* to remove bacterial cells. Phage titer in the supernatant was determined by plating 10-fold dilutions on the plates with precast 1.2% bottom LB agar and 0.6% top LB agar mixed with a 100-fold diluted BW25113 overnight culture. Plates were stored at 37 °C overnight. The experiment was performed in biological triplicate.

### 2.6. Liquid Culture Infection

To monitor the dynamics of Sxt1 and T7 phage infection in liquid, we used an EnSpire Multimode Plate Reader (Perkin Elmer, Hongkong, China). Overnight bacterial cultures were diluted 100-fold in LB medium at 37 °C in 10 mL of LB. At OD_600_ = 0.6 (~2 × 10^8^ cfu/mL), 200 μL aliquots were transferred to 96-well plates and infected at indicated MOI using a 10^10^ pfu/mL phage stock. Optical density was monitored for 3 h. All experiments were performed in three biological replicates.

### 2.7. Determination of the Host Range

To determine the host range of Sxt1, T7, and T3, the efficiency of the plating assay was conducted using the double agar overlay method. Overnight cultures of bacteria (100 µL) were mixed with 10 mL of 0.6% top LB agar and poured on the surface of precast 1.2% bottom LB agar plates. A volume of 3 µL drops of serial 10-fold phage (10^10^ pfu/mL stock) lysate dilutions were spotted on the top agar, allowed to dry, and plates were incubated at 37 °C overnight. 

### 2.8. Adsorption Assay

An adsorption assay was performed with *E. coli* ECOR50 strain. Cell culture was grown in LB at 37 °C until OD_600_ = 0.6 (~2 × 10^8^ cfu/mL). Sxt1, T7, and T3 from a 10^10^ pfu/mL stock were added to the aliquots of bacterial culture to achieve MOI of 0.001. The 1 mL aliquots were immediately taken from each replicate to estimate the initial phage titer. The second aliquot was taken after 7 min incubation at room temperature. The aliquots were centrifuged for 5 min at 6000× *g* and phage titer in the supernatant was determined via the plating on BW25113 cell lawns, as described above. Adsorption efficiency was calculated as 1—(pfu at 7 min/pfu at 0 min).

### 2.9. DNA Sequencing

Libraries for shotgun sequencing were prepared from 500 ng of phage DNA using MGIEasy Universal DNA Library Prep Set (MGI Tech, Shenzhen, China), following the manufacturer’s instructions. DNA was sonicated using a Covaris S-220 followed by selection of 200–250 bp-long fragments on magnetic beads (MagBio, Gaithersburg, MD, USA). The concentration of the prepared libraries was measured using Qubit Flex (Life Technologies, Carlsbad, CA, USA) with the dsDNA HS Assay Kit. The quality of the prepared libraries was assessed using a Bioanalyzer 2100 with the High Sensitivity DNA kit (Agilent, Santa Clara, CA, USA). DNA libraries were further circularized and sequenced by a paired end sequencing using DNBSEQ-G400 with the High-throughput Sequencing Set PE100, following the manufacturer’s instructions (MGI Tech), with an average coverage of 100×. FastQ files were generated using the zebracallV2 software (MGI Tech).

### 2.10. Phage Genome Assembly and Annotation

At the first stage, fastp v. 0.23.2 was used to trim adapters, deduplicate raw reads, and check quality [32]. Unicycler v. 0.5.0 was used to assemble a genome with k-mer size of 81 and normal bridges connection mode [33]. The closest sequence similarity of assembled phage to the T3 was established with the help of whole-genome blastn search against non-redundant sequences [34]. Whole-genome alignment of selected sequences with Sxt1 was performed with web VIRIDIC [35]. The genome start position was manually assigned by an alignment to the genome of phage T3. fastANI tool v. 1.33 with fragment length parameter equal to 150 was used to establish nucleotide sequence similarity [36]. Phage genome annotation was performed using pharokka v. 1.3.1. [37]. Coding sequences were predicted with PHANOTATE v.1.5.1 [38]. Functional annotation was performed based on the following databases: PHROGs v.4 [39], VFDB [40], and CARD v.3.2.7 [41], with the help of MMseqs2 v.13.45111 [42] and PyHMMER v.0.10.5 [43]. Resulting contigs were matched to their closest hit in the INPHARED database [44] using mash v.2.3 [45]. Manual refinement of predicted hypothetical coding sequences was performed, and all entries which shared high nucleotide sequence identity with the phage T3, yet do not correspond to the known CDSs, were considered as false positives and were removed.

### 2.11. Sequence Alignment, Phylogenetics Analysis, and Structural Comparison

Based on the Sxt1 whole-genome blastn hits, 13 phages from different genera (*Berlinvirus*, *Pifdecavirus*, *Przondovirus*, *Teetrevirus*, *Teseptimavirus*, and *Unyawovirus*) representative of the *Autographiviridae* family were selected.

To build a phylogenetic tree, the orthogroups of 14 phages under analysis were established with Orthofinder v. 2.5.5 [46]. As a result, 43 protein orthogroups were used to build MSA with MAFFT v. 7.520 [47]. IQ-TREE2 v. 2.2.2.3 [48] was used to build maximum-likelihood phylogenetic tree with the following parameters: the Q.pfam + F + I + R3 substitution model for amino acid sequences was selected with ModelFinder [49], and an ultrafast bootstrap procedure of 10,000 replicates was set to evaluate support (-B 10,000) [50].

An identical procedure was applied to the protein sequences, which were not similar to orthologues of the T3 phage: internal virion protein (sxt1_p36, homolog of T3 gp15), internal virion protein with endolysin domain (sxt1_p37, homolog of T3 gp16), and lateral tail fiber protein (sxt1_p38, homolog of T3 gp17) with LG + F + G4, LG + F + G4, and Blosum62 + F+I + G4 substitution models, correspondingly.

In order to compare the protein structure of the tip-domain of Sxt1 lateral tail fiber protein (sxt1_p38) with the known X-ray structure of the phage T7 gp17 C-terminal domain trimer [51], ColabFold v.1.5.2-patch [52] was used for the prediction of the Sxt1 gp17 homotrimer structure (residues 502-667).

### 2.12. Visualization of the Results

R v. 4.2.3 with library ‘tidyverse’ v. 2.0.0 was used to visualize genetic maps (library ‘gggenomes’ v. 0.9.9.9000) and to plot phylogenetic trees (libraries ‘ggpubr’ v. 0.6.0, ‘ggtree’ v. 3.9.1, ‘phytools’ v. 1.9-16). Python v. 3.11, Pymol v.2.5.0 were used to visualize protein structures, and package ‘pymsavis’ v. 0.4.0 was used to visualize multiple sequence alignment.

## 3. Results and Discussion

### 3.1. Isolation, Life Cycle, and Morphology of Sxt1

**Figure 1:**
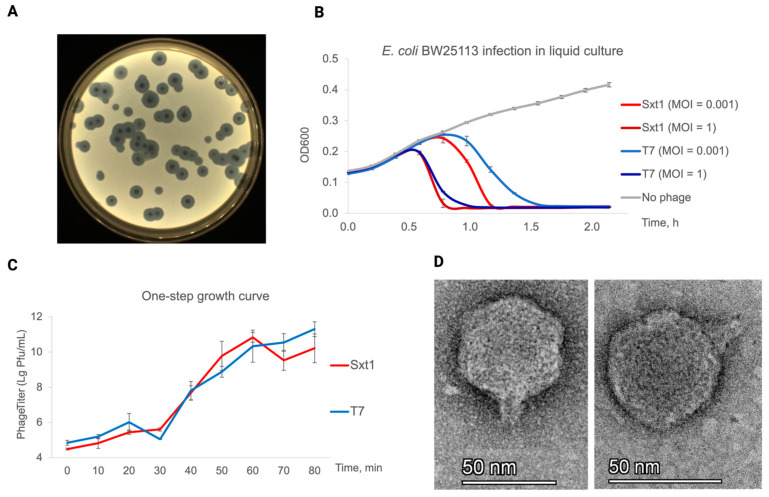
(**A**) Morphology of Sxt1 plaques on the *E. coli* BW25113 cell lawn. (**B**) Infection of *E. coli* BW25113 liquid cultures with Sxt1 or phage T7 at low (0.001) or high (1) MOI. (**C**) One-step growth curve of Sxt1 and T7 phages in liquid LB media at 37 °C. (**D**) Representative TEM images of the Sxt1 virions.

Using polyvalent phage cocktail Sextaphage^®^ (Microgen, Perm, Russia) as a source of uncharacterized phages claimed to have established therapeutic potential [25,28], we isolated a series of coliphages using *E. coli* BW25113 as a host. Phages have been serially re-streaked from single plaques to avoid cross-contamination. One of the phages, hereafter named Sxt1, produced quickly growing large clear plaques, resembling those formed by the *Autographiviridae* family phages (Figure 1A). Similarly to the prototypical podovirus T7, Sxt1 lysed BW25113 cultures in ~20 min at 37 °C and had a burst size of ~100 progeny particles (Figure 1B,C). TEM imaging confirmed the podovirus-like morphology of the virion: Sxt1 has an icosahedral capsid (a mean of 55 nm ± 5 nm in diameter) with a short non-contractile tail (a mean of 19 ± 3 nm in length) and lateral tail fibers, which are poorly resolved on TEM images (Figure 1D).

### 3.2. Genomic Organization of the Sxt1

Sxt1 has a linear 39,368 bp dsDNA genome that is highly similar to the T3 phage genome (Table 1). The average genomic GC content is 50.45%. Although PhageTerm [53] did not confidently predict the type of Sxt1 terminal repeats, 227 bp-long direct terminal repeats were readily identified through alignment with the T3 phage genome. Sxt1 genes were predicted with Pharokka [37] and manually curated, revealing the presence of 46 open reading frames (Table S1). No tRNA genes and genes encoding known toxins (according to the VFDB reference database of bacterial virulence factors [40]) were identified. In total, 42 out of the 46 Sxt1 ORFs demonstrate high sequence identity with the corresponding T3 ORFs (Table S1).

**Figure 2:**
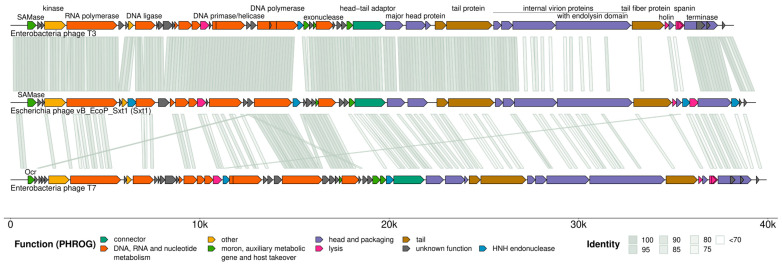
Genomic comparison of Sxt1, T3, and T7 phage genomes. Colors represent functional annotations according to the PHROG database. Predicted HNH endonucleases genes are marked in blue. Sequence identity was calculated using fastANI with a 150 bp window. Genetic maps were prepared using gggenomes library in R.

Overall, the Sxt1 genome demonstrates complete colinearity with the genomes of the model coliphages T3 and T7, belonging to the *Autographiviridae* family (Figure 2). A signature feature of the *Autographiviridae* phages is the viral RNA polymerase that transcribes the phage genome, except for the pre-early genes, which are located upstream of the viral RNA polymerase gene and transcribed by host RNA polymerase. Transcription of pre-early genes by host RNA polymerase facilitates injection of the phage genome [54]. When transcription by viral RNA polymerase commences, host transcription is shut-off [55]. Pre-early genes encode host-takeover and anti-defense proteins, such as SAM lyase (T3) or DNA mimic Ocr (T7), that inhibit BREX and Type I Restriction-Modification defenses of the host [56,57,58]. The gp0.7 kinase of both T7 and T3 phosphorylates a wide range of cellular proteins, including host RNA polymerase, and is involved in the host transcription shut-off and host immunity suppression [59,60]. The pre-early region of Sxt1 is almost completely identical with the phage T3 at the nucleotide level.

The *Autographiviridae* early genes are involved in DNA metabolism and phage genome replication, while the late genes encode structural components of phage virions, genome packaging (terminase), and host lysis (holin and spanins) [61]. Sxt1 in the early and part of the late genomic regions also demonstrate a high level of identity with the T3 genome. In contrast, genes encoding lateral tail fiber and two internal virion proteins (sxt1_p36–38, homologs of T3 gp15, gp16, and gp17) are quite distinct and must have been acquired through a recombination event with a different phage. These proteins will be discussed in more detail below. In addition, Sxt1 has experienced expansion of homing HNH nuclease genes with four members of this family that are not present in T3 or T7 genomes dispersed along its genome (Figure 2), which accords with their “parasitic” nature [62].

### 3.3. Comparative Genomics of Sxt1

**Figure 3:**
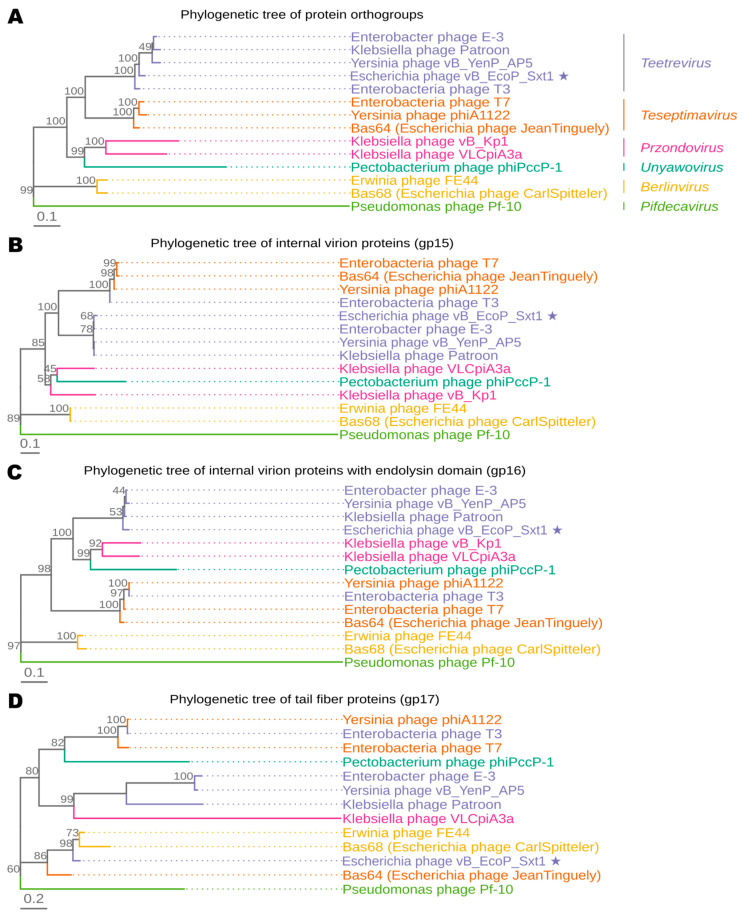
The position of Sxt1 on the phylogenetic tree of bacteriophages from the *Studiervirinae* subfamily. (**A**) Phylogenetic tree of indicated bacteriophages based on protein orthogroups, except those analyzed in panels (**B,C**). Phylogenetic trees of internal virion proteins gp15 (sxt1_p36) (**B**), internal virion proteins with endolysin domains gp16 (sxt1_p37) (**C**), and lateral tail fiber proteins gp17 (sxt1_p38) (**D**). Bacteriophage genera are indicated with color. Bootstrap support is provided at each node. Branch lengths reflect the numbers of substitutions per site. Sxt1 position is indicated with a star.

According to the blastn whole-genome search, the closest relatives of Sxt1 are Klebsiella phages vB_KpnP_Emp27 (88% genome coverage, 93.94% identity, GenBank id—MN013074.1), KPP-5 (87% genome coverage, 94.10% identity, GenBank id—MW600722.1), phage Patroon (85% genome coverage, 93.82% identity, GenBank id—MK608335.1), and the T3 phage (77% genome coverage, 92.79% identity, GenBank id—NC_003298.1) [63,64,65]. These first three phages infect diverse multi-drug-resistant *K. pneumonia* strains. We performed whole-genome alignment of Sxt1 against selected members of the *Teetrevirus*, *Teseptimavirus, Przondovirus, Unyawovirus, Berlinvirus*, and *Pifdecavirus* genera using VIRIDIC (Figure S1). In addition, we constructed a phylogenetic tree of these phages using orthogroups of 43 conserved proteins (see Section 2) (Figure 3A). Sxt1 clustered with other *Teetrevirus* phages with a confident bootstrap support and thus represents a novel specie of this genus. According to the proposed phage naming nomenclature [66], the phage received the name vB_EcoP_Sxt1. For simplicity, we will continue to use a shorter name, Sxt1.

Next, we focused on the most divergent Sxt1 proteins (sxt1_p36–38), homologs of T3 gp15, gp16, and LTF (gp17). Phylogenetic trees for each of these proteins were not co-linear with the overall phage genomes phylogeny (Figure 3B–D). The gp14, gp15, and gp16 are internal virion proteins that comprise ejectosome, a molecular machine that protrudes through the outer membrane, lyses the peptidoglycan layer with the N-terminal gp16 hydrolase domain, and then forms a channel across the periplasm for phage genome injection into the host cell [29,30,67]. A large toroid-shaped C-terminal vinculin-like domain of gp16 is inserted into the cytoplasm and may serve as a motor for phage genome injection [68]. The gp15 (sxt1_p36, a channel-forming component) and gp16 (sxt1_p37, protein with hydrolase and vinculin-like domains) of Sxt1 clustered with other *Teetrevirus* members, while the T3 homologs were closer to representatives of *Teseptimavirus* and phage T7 (Figure 3B,C). This suggests that the phage T3 experienced a recombination event with genomic locus-encoding ejectosome components of a *Teseptimavirus.*

The lateral tail fiber protein (LTF) of Sxt1 did also not cluster with the orthologs of other *Teetrevirus* members, including T3, but showed greater similarity to LTFs of the *Berlinvirus* phages (Figure 3D). This is particularly intriguing given that Sxt1 was isolated on the *E. coli* BW25113 host, which is not susceptible to T3 phage infection [69]. This observation prompted us to further examine the differences in LTF organization of T3 and Sxt1 and compare their host ranges.

### 3.4. Sxt1 Encodes Extended Lateral Tail Fibers

*Autographiviridae* phages exploit lipopolysaccharides (LPS) for initial reversible attachment to the bacterial cell surface using six tail fibers, each composed of a trimer of the LTF protein gp17 (sxt1_p38) [51,67]. T7 virions can even “walk” on the surface of the cell before irreversible attachment facilitated by other tail proteins [67]. The host specificity of *Autographiviridae* is mediated by specific HRDRs (host range-determining regions) on the LTF tip, which contacts the cell surface [70]. The alignments of the Sxt1, T3, and T7 phages’ LTFs are presented in Figure S2. The N-terminal LTF domain is required for attachment to the tail tube and is conserved between the three phages. The long pyramid domain of LTF is formed by an intertwined beta-layer, which includes beta-sheets from each monomer in the assembled trimer [51,70]. It is followed by a short alpha-helical linker and the tip domain responsible for the interaction with LPS [51,70]. We noticed that compared to T3 and T3, the pyramid domain of Sxt1 contained six insertions of more than five amino acids in length, some of which could form additional beta-sheets. The alpha-helical linker also contained a 27 amino-acid insertion. These inserts should extend the overall length of the Sxt1 tail fiber (Figure S2).

**Figure 4:**
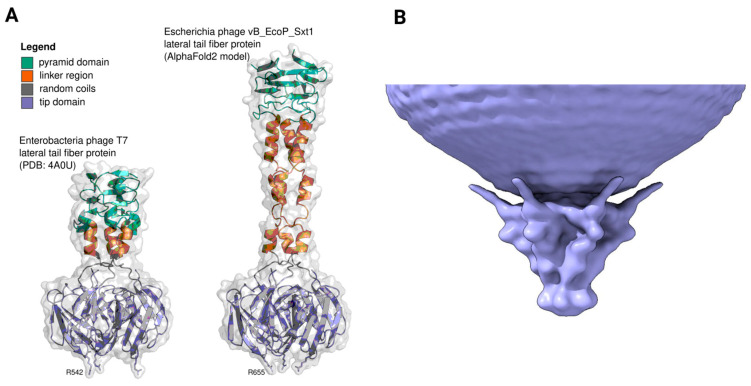
(**A**) C-terminal receptor-sensing domains of lateral tail fiber protein homotrimer from phage T7 (PDB: 4A0U, left) and Sxt1 (AF2 model, right). (**B**) Reconstruction of the Sxt1 virion, revealing the structure of the tail and positions of the six lateral tail fiber attachment sites. The model was obtained using a C6 symmetry axis.

We attempted to model the Sxt1 LTF trimer using AlphaFold2; however, the complex structure of the pyramid domain hindered predictions. We therefore next modeled only the tip domain with an alpha-helical linker and a small part of the pyramid domain, which allowed us to compare the Sxt1 LTF with a published structure of T7 LTF (Figure 4A). The Sxt1 LTF tip domain shares 43% identity with T3 and 54% with T7 (Table 1). It preserves the same fold, with HRDRs exposed at the bottom of the protein. We also noted that R655 protrudes from the tip of the LTF, suggesting its role in the interaction with the cell’s surface, and its position differs between T3/Sxt1 and T7, suggesting the recognition of a different set of host primary receptors (Figure 4A and Figure S2).

Driven by these observations, we attempted to obtain a high-resolution structure of the Sxt1 virion, and collected a cryo-electron microscopy dataset, which allowed us to obtain a reconstruction with an average 16 Å resolution (Figure 4B and Figure S3A,B). The virion model resolves the Sxt1 tail and N-terminal domain of the six LTFs, protruding in the direction of the capsid. The LTF CTD was not resolved in our structure, suggesting conformational heterogeneity and flexibility in this region, in accordance with other reconstructions of podovirus-like virions [71,72,73]. A lateral cross-section of the Sxt1 virion demonstrates a low-resolution density, which can be attributed to LTFs and suggests that they are not tightly attached to the capsid and thus could be mobile (Figure S3C).

### 3.5. Sxt1 Has a Broader Host Range Compared to T3 and T7 Phages

**Figure 5:**
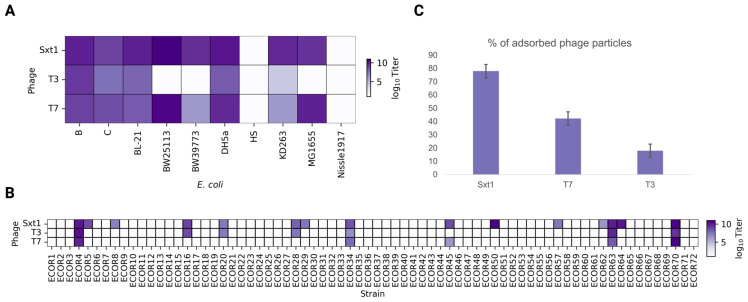
(**A**) Efficiency of Sxt1, T3, and T7 plaquing on common *E. coli* strains. (**B**) Efficiency of Sxt1, T3, and T7 plaquing on a collection of natural *E. coli* isolates producing O-antigens. (**C**) An adsorption assay with *E. coli* ECOR50 and Sxt1, T3, and T7. % of phage particles adsorbed on the surface of bacterial cells 7 min after infection is shown.

We further characterized the host specificity of Sxt1. We plated Sxt1, T7, and T3 phages on widely used *E. coli* strains BW25113, MG1655, BL-21, B, C, DH5α, HS, and Nissle1917, and on F+ strains BW39773 and KD263, which should inhibit T7 due to the presence of an abortive infection system, PifA [74]. While the two common gut commensals, HS and Nissle1917 [75], were resistant to all phages, other *E. coli* strains were infected by Sxt1 (Figure 5A). T3 was unable to infect strains of the K-12 lineage, while T7 was suppressed by hosts with the PifA system.

Most laboratory strains of *E. coli* K-12 do not produce O-antigens due to transposon insertion in the *wbbL* gene [76,77]. Contrariwise, most environmental and clinical isolates of *E. coli* encode O-antigens. At least 185 types of *E. coli* O serogroups are recognized, and O-antigens could represent one of the major barriers for host recognition by phages [77,78,79]. Thus, phages with extended host ranges and O-antigen tolerance/specificity should be better candidates for phage therapy applications. To investigate whether atypical structures of Sxt1 LTF confer broader host specificity and an ability to infect O-antigen-carrying hosts, we compared Sxt1, T3, and T7 plaquing efficiency using the reference ECOR host collection, representing the natural diversity of *E. coli* strains [80]. Sxt1 was able to infect 15 out of 72 strains in this collection (20%), which included all hosts susceptible to T7 or/and T3, and an additional 7 ECOR strains that were resistant to these phages (Figure 5B). Matching known ECOR O-antigens with Sxt1 sensitivity did not reveal a preference for a specific O-antigen type [81], and bacterial hosts encoding different core oligosaccharide types (K12, R1–R4) were infected, highlighting the fact that subtle differences in O-antigen chemical structure could have a drastic effect on phage infectivity and, at the same time, O-antigens with different core structures can be targeted by the same phage.

The inability of a phage to infect a host cell can be associated not only with receptor recognition, but also with the presence of anti-phage immunity systems [79]. We selected strain ECOR50, which is sensitive to Sxt1 but not to T7 or T3, and performed an adsorption efficiency assay, which confirmed that only Sxt1 efficiently bound to the cell surface (Figure 5C). Thus, the expanded host range of the Sxt1 is likely explained through receptor recognition and not an enhanced resistance to the immunity systems.

## 4. Conclusions

With the desperate need to find novel solutions for the antibiotic resistance crisis, great effort has been put into the development of phage therapy. However, this field also faces many challenges, including narrow specificity of the phages to bacterial receptors and diversity of the cell surface structures, blocking phage binding. Therefore, phages with a broader host range carry a greater potential for practical applications, and estimation of the O-antigen specificity/tolerance could become a standard procedure for phage characterization. Here, we report on Sxt1, a novel *Teetrevirus* closely related to phages T3 and T7, isolated from a therapeutic phage cocktail Sextaphage^®^. Differences between these phages are summarized in Table 1. We provide the genomic sequence of the Sxt1 and characterize its host range on an ECOR collection of natural *E. coli* isolates with divergent O-antigen types. The phage demonstrates an extended host range compared to T3 and T7 relatives. According to previous studies, this difference could be attributed to the variability in host range determining regions of the LTFs [70], although the extended length of the Sxt1 LTFs also might contribute to the receptor recognition. Of note, LTFs of the Sxt1 were likely acquired from a *Berlinvirus* phage. Lytic *Autographiviridae* phages that have an “aggressive” lifestyle, i.e., quick lysis time and large phage bursts, represent promising agents for phage therapy. Our research warrants further use of Sxt1 as a therapeutic agent against *E. coli* infections and the need for the investigation of individual phages and phage cocktails in clinical settings and against hosts with diverse cell surface organization.

## Figures and Tables

**Table 1:** Genetic features and host range of the phages Sxt1, T3, and T7.

	Feature	Sxt1	T3	T7
Phage				
Genome length, bp		39,368	38,208	39,937
GC-content, %		50.5	50	48.5
# CDSs *		46	47	53
DTR length, bp		227	231	160
Host range **		15/72	7/72	5/72
LTF tip domain identity, %		100	43	54
LTF protein length, aa		667	558	553

* CDS were determined as described in Materials and Methods. ** Proportion of the *E. coli* ECOR strains infected by a given phage, discussed below.

## Data Availability

Complete genome of the Sxt1 has been deposited to the GenBank under accession number OR545379.1. Sxt1 and other materials used in this work are available upon request from the lead contact, Dr. Artem Isaev.

## Supplementary Materials

The following supporting information can be downloaded at: https://www.mdpi.com/article/10.3390/v16121905/s1, Table S1: Sxt1 predicted ORFs and their sequence identity to homologs from Enterobacteria phage T3; Figure S1: Sxt1 shares highest intergenomic similarities with phages from *Teetrevirus* genus; Figure S2: Multiple sequence alignment of lateral tail fiber protein sequences under study; Figure S3: Cryo-EM investigation of the Sxt1 virion structure.
