# Supplementary material for: Sxt1, Isolated from a Therapeutic Phage Cocktail, Is a Broader Host Range Relative of the Phage T3
Source: Viruses. 2024 Dec 11;16(12):1905. doi: 10.3390/v16121905 (PMC11680406; doi:10.3390/v16121905)
Supplement: Supplementary file 1 [file viruses-16-01905-s001.zip › viruses-3313338-supplementary.pdf]

Table S1. Sekstaphage-1 predicted ORFs and their sequence identity to homologs from Enterobacteria phage T3.

ORF	Start	End	Length (bp)	Molecular mass (kD)	Identity to T3 homolog	T3 homolog	Product
1	910	1368	458	17.0	1.0	gp0.3	SAMase
2	1439	1636	197	7.7	1.0	gp0.5	hypothetical protein
3	1668	1790	122	4.7	0.73	gp0.6	hypothetical protein
4	1805	2914	1109	42.5	0.94	gp0.7	serine-threonine kinase
5	2973	5639	2666	99.2	0.99	gp1	RNA polymerase
6	5747	5887	140	5.9	0.98	gp1.05	hypothetical protein
7	5890	6162	272	10.5	0.91	gp1.2	dGTPase inhibitor; target for F exclusion
8	6201	6656	455	17.8	[1]0.13	-	HNH endonuclease
9	6628	7644	1016	38.4	0.9	gp1.3	DNA ligase
10	7812	8381	569	21.8	0.43	gp1.8	hypothetical protein
11	8432	8665	233	8.8	0.65	gp2	RNA polymerase inhibitor
12	8718	9416	698	25.9	0.99	gp2.5	Gp2.5-like ssDNA binding protein and ssDNA annealing protein
13	9416	9877	461	17.6	0.95	gp3	endonuclease
14	9870	10325	455	16.9	1.0	gp3.5	amidase
15	10336	10437	101	3.9	0.94	gp3.7	hypothetical protein
16	10503	12203	1700	62.7	1.00	gp4A	DNA primase/helicase
17	12299	12511	212	7.7	0.99	gp4.3	hypothetical protein
18	12524	12808	284	10.7	1.0	gp4.5	hypothetical protein
19	12876	14858	1982	74.9	0.93	gp5	DNA polymerase I
20	14920	15315	395	15.2	0.36	gp5.3	HNH endonuclease
21	15478	15591	113	4.3	-	-	hypothetical protein
22	15611	15910	299	11.1	0.49	gp5.5	hypothetical protein
23	15910	16119	209	7.4	0.99	gp5.7	hypothetical protein
24	16119	16277	158	6.1	1.0	gp5.9	Gp5.9-like inihibitor of recBCD nuclease
25	16264	17172	908	34.6	1.0	gp6	exonuclease
26	17360	17605	245	9.3	0.95	gp6.5	hypothetical protein
27	17610	17858	248	8.8	0.92	gp6.7	hypothetical protein
28	17885	18178	293	10.1	0.89	gp7.3	host range and adsorption protein
29	18189	19796	1607	58.7	0.99	gp8	head-tail adaptor
30	19897	20829	932	33.7	0.97	gp9	head assembly
31	20986	22023	1037	36.6	0.98	gp10A	major head protein
32	22520	23110	590	22.2	0.99	gp11	tail protein
33	23126	25531	2405	90.0	0.98	gp12	tail protein
34	25604	26014	410	15.8	0.96	gp13	internal virion protein
35	26017	26610	593	21.1	0.99	gp14	internal virion protein
36	26613	28856	2243	85.0	0.69	gp15	internal virion protein
37	28875	32837	3962	143.5	0.66	gp16	internal virion protein with endolysin domain
38	32917	34920	2003	73.5	0.34	gp17	tail fiber protein

39	34968	35174	206	7.3	0.84	gp17.5	holin
40	35167	35436	269	10.2	0.74	gp18	terminase small subunit
41	35495	35896	401	15.0	[1]0.18	-	HNH endonuclease
42	35877	36332	455	16.9	0.51	gp18.5	Rz-like spanin
43	36307	38070	1763	66.7	0.97	gp19	terminase large subunit
44	38080	38514	434	16.6	[1]0.17	-	HNH endonuclease
45	38495	38608	113	4.3	0.18	gp19.3	hypothetical protein
46	38737	38886	149	5.5	0.78	gp19.5	hypothetical protein

[1] Compared with gp5.3 HNH endonuclease.

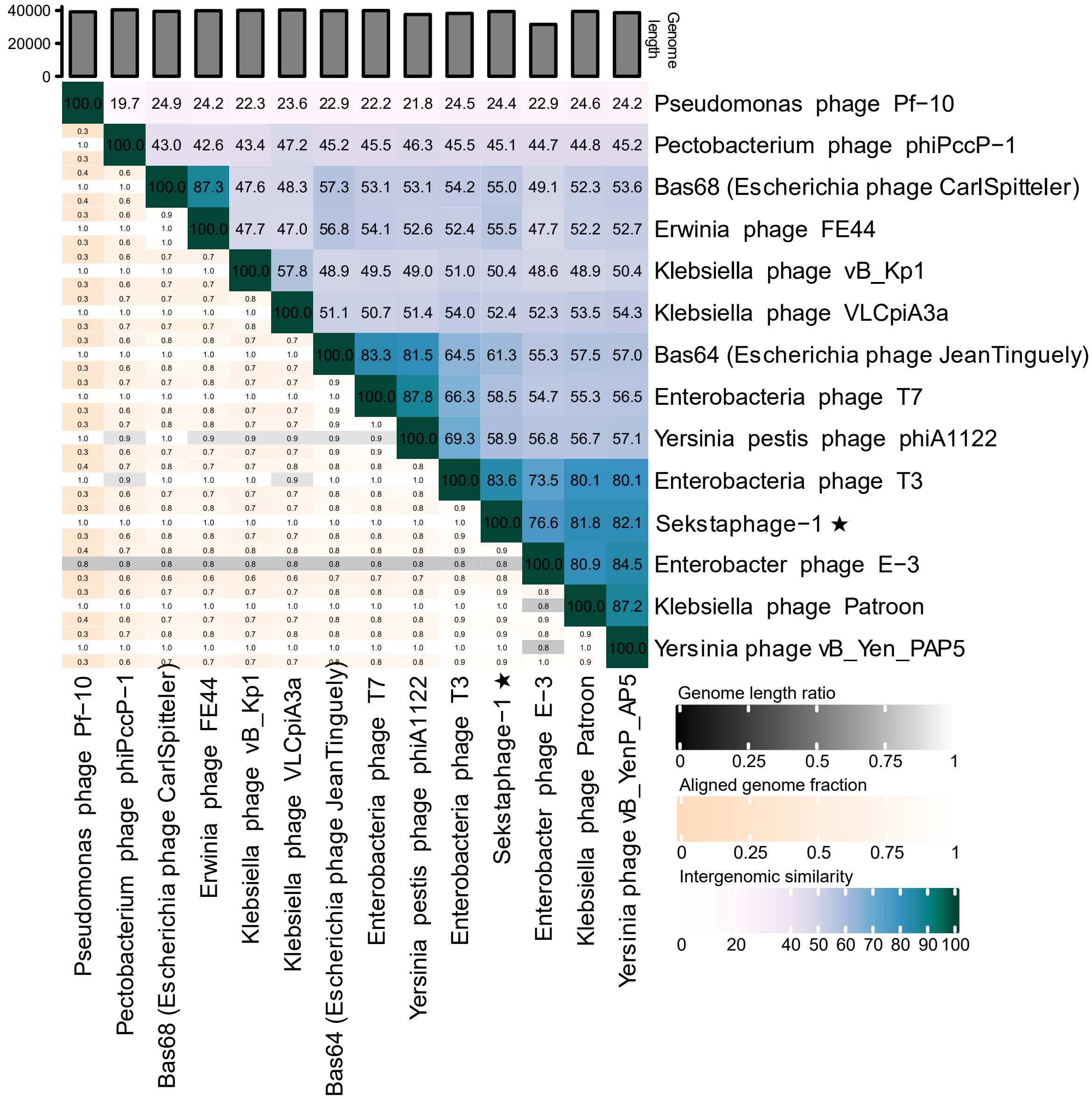

Figure S1. Sekstaphage-1 shares highest intergenomic similarities with phages from *Teetrevirus* genus. Pairwise genomic similarities amongst Sekstaphage-1 closest relatives, computed with VIRIDIC. Sekstaphage-1 marked with a star (★).

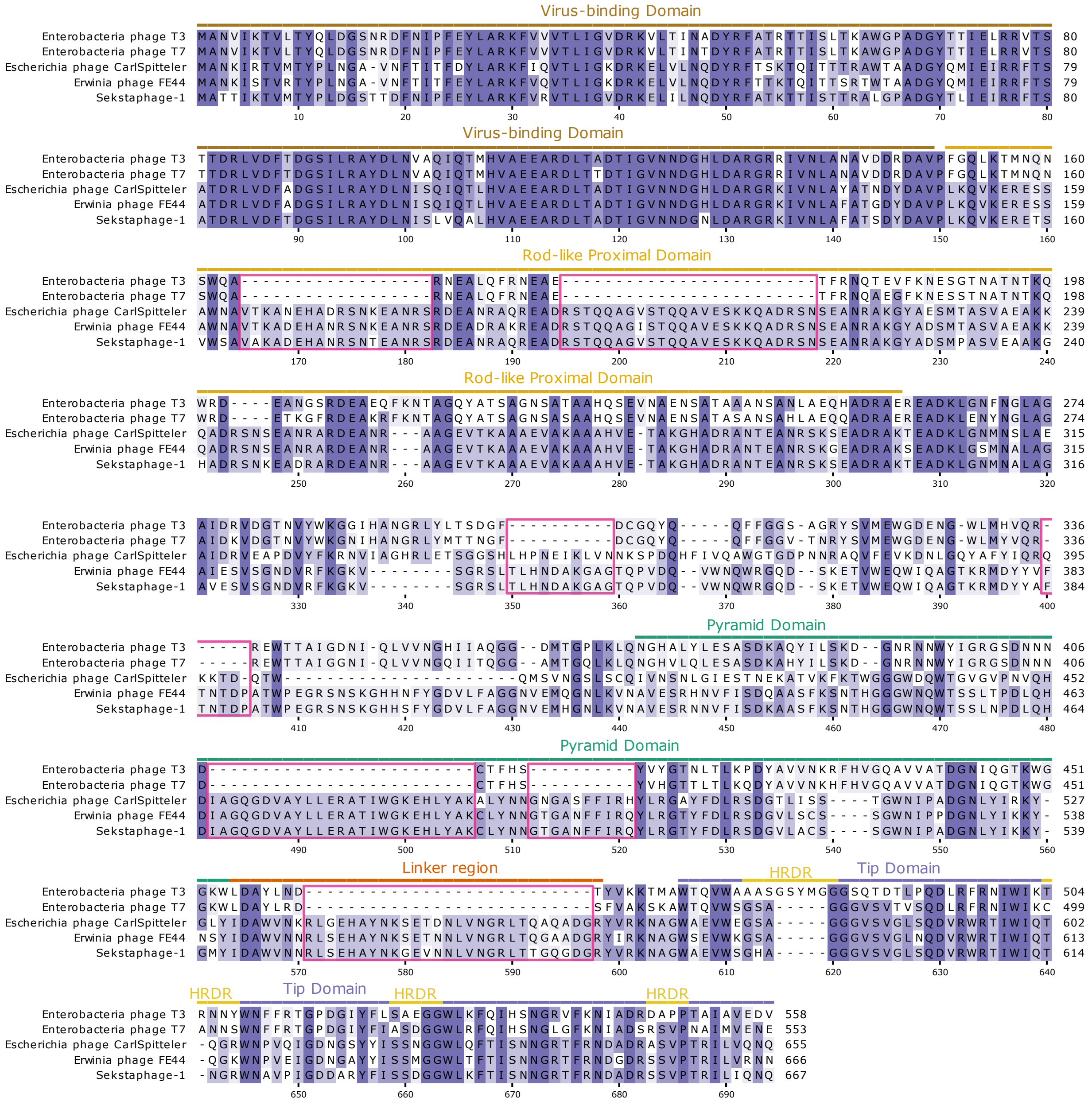

Figure S2. Multiple sequence alignment of tail fiber protein sequences (gp17) under study. Conservation of amino acids positions is shown by color. Insert regions longer than 5 amino acids are marked by pink rectangles. The known domains and HRDR regions are shown by colors.

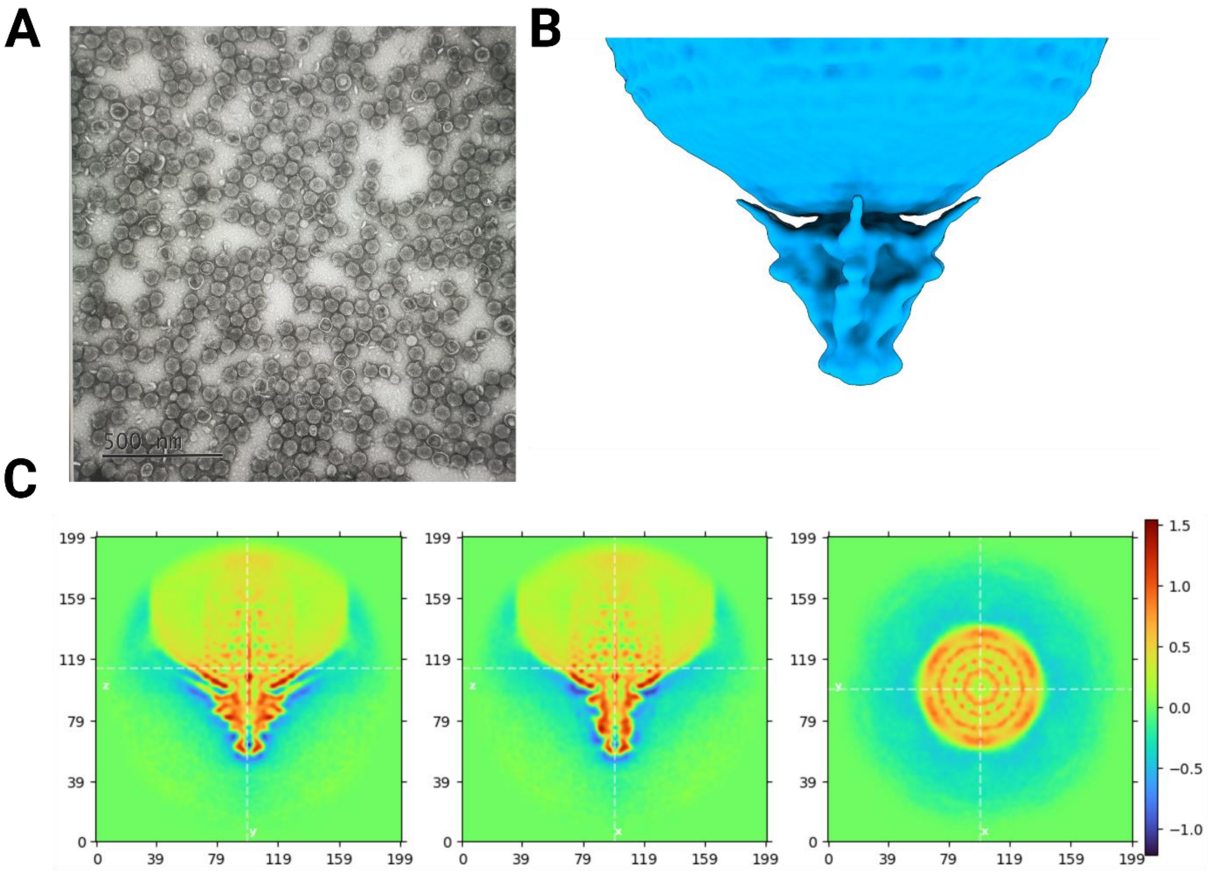

Figure S3. Cryo-EM investigation of the Sekstaphage-1 virion structure. **(A)** A representative field of view demonstrating phage virions subjected to analysis. **(B)** A model of the Sekstaphage-1 tail obtained with an asymmetric reconstruction (~20 Å resolution). **(C)** A lateral and radial cross-section of the Sekstaphage-1 virion, highlighting a possible position of the LTFs.
